# The Variable Influence of Orthotic Management on Hip and Pelvic Rotation in Children with Unilateral Neurogenic Equinus Deformity

**DOI:** 10.3390/children10020307

**Published:** 2023-02-06

**Authors:** Domenic Grisch, Manuela Stäuble, Sandra Baumgartner, Hubertus J. A. van Hedel, Andreas Meyer-Heim, Thomas Dreher, Britta Krautwurst

**Affiliations:** 1 Department of Pediatric Orthopedics, Neuroorthopedics and Traumatology, University Children’s Hospital Zurich, 8032 Zurich, Switzerland; 2 Swiss Children’s Rehab, University Children’s Hospital Zurich, 8910 Affoltern am Albis, Switzerland; 3 Pediatric Orthopedics, Balgrist University Hospital, University of Zurich, 8008 Zürich, Switzerland

**Keywords:** hemiplegia, cerebral palsy, pelvic asymmetry, hip asymmetry, pes equinus, orthotic management

## Abstract

Background: Equinus deformity with or without concomitant drop foot is a common finding in children with unilateral spastic cerebral palsy and spastic hemiplegia of other causes. Hypothetically, these deformities may lead to pelvic retraction and hip internal rotation during gait. Orthoses are used to reduce pes equinus during gait and to restore hindfoot first contact. Objective: We aimed to investigate whether the use of orthotic equinus correction reduces rotational hip and pelvic asymmetries. Methods: In a retrospective study, 34 children with unilateral spastic cerebral palsy or spastic hemiplegia of other causes underwent standardized instrumented 3D gait analysis with and without orthotic equinus management. We analyzed the differences in the torsional profile during barefoot walking and while wearing orthoses, as well as investigated the influence of ankle dorsiflexion and femoral anteversion on pelvic and hip kinematics and hip kinetics. Results: Wearing orthoses corrected pes equinus and pelvic internal rotation at the end of the stance phase and in the swing phase compared to barefoot walking. Hip rotation and the rotational moment did not significantly change with orthoses. Orthotic management or femoral anteversion did not correlate to pelvic and hip asymmetry. Conclusion: The findings indicate that the correction of the equinus by using orthoses had a variable effect on the asymmetry of the hip and pelvis and internal rotation; both appear to have a multifactorial cause that is not primarily driven by the equinus component.

## 1. Introduction

The most common gait abnormalities in children with unilateral spastic cerebral palsy and spastic hemiplegia of other causes are pes equinus with or without drop foot, in-toeing, and stiff knee in the swing phase. Other manifestations are hip flexion, hip internal rotation, and pelvic retraction [1,2,3]. As a consequence of these abnormalities, children are prone to developing lever arm dysfunction and clearance problems, such as dripping and falling [1]. The standard treatment for in-toeing gait is femoral derotation osteotomy (FDO). The surgically improved hip centration results in reduced internal rotation of the hip and pelvic retraction [4,5]. Nevertheless, FDO carries the risk of over- or under-correction of rotational abnormalities [6]. To date, the pathogenetic mechanisms of internal rotation gait are not fully understood, and dynamic factors need to be discussed. Dynamic components can include muscular imbalance, increased muscle tone, spasticity, and altered moment arms [7]. It was hypothesized that hip internal rotation might be a result of high femoral anteversion or increased hip flexion present in children with unilateral spastic cerebral palsy [8,9]. However, it is still unclear whether this is a cause or a consequence of other factors. This might explain the variability of results after femoral derotation osteotomies. Based on computerized modeling, Brunner et al. showed a significant correlation between ankle plantar flexion and hip internal rotation in children with unilateral spastic cerebral palsy as a direct functional effect. Hence, an equinus position changes the torsional forces in the hip joint and, thus, increases hip internal rotation and pelvic retraction. This mechanism may represent a driving factor for the persistence of increased femoral anteversion during growth. Furthermore, it was suggested that the effects at the hip and knee are related to the function of the triceps surae and are not directly dependent on neuromuscular control [10]. In addition, Pasin Neto et al. found a combined improvement of ankle dorsiflexion and internal rotation of the hip through postural insoles in children with bilateral spastic cerebral palsy [11]. However, the body of literature lacks studies that underline the hypothesis that increased plantar flexion is a relevant factor leading to internal rotation gait.

Orthotic management is typically applied to correct pes equinus during walking and restore heel contact at initial contact (IC) in children with unilateral spastic cerebral palsy or spastic hemiplegia of other causes [12,13,14]. This results in an improvement of the first and second ankle rocker, increased dorsiflexion, and reduced drop foot [12,13,15,16,17]. Furthermore, orthotic management to correct pes equinus decreases energy cost, increases speed and stride length, enlarges hip and ankle range of motion, and improves the kinematics and kinetics of the knee [12,15,16,17,18,19]. 

In addition to conservative treatment with orthotics or the surgical rotation correction of the femur described above, various other treatment approaches are used for spasticity management, the improvement of foot elevation, or the correction of contractures. These include oral spasmolytics, selective dorsal rhizotomy, oral or intrathecal baclofen [20], botulinum toxin injections [21], muscular strengthening and stretching through physiotherapy, neuromuscular electrical stimulation [22], lengthening of the calf muscles, and shortening of the anterior tibialis tendon with or without split transfer to peroneus brevis [23]. The latter contributes to the balanced, active foot elevation or at least leads to a supporting tenodesis.

If pes equinus plays a central role as a driving factor for internal rotation gait, torsional moments of the hip, hip internal rotation, and pelvic asymmetry [1,2,3,10,24], these patterns should vanish or at least be significantly reduced by the orthotic management of equinus foot deformity. However, to the best knowledge of the authors, this was not previously investigated.

We hypothesized that the rotational effects and asymmetry of the hip and pelvis are significantly reduced by using orthoses to correct pes equinus in children with unilateral spastic cerebral palsy and spastic hemiplegia of other causes. As a secondary research question, this study aimed to investigate whether a high femoral anteversion reduces the corrective effect of the orthoses on hip and pelvic asymmetry.

## 2. Materials and Methods

### 2.1. Participants

**Figure 1:**
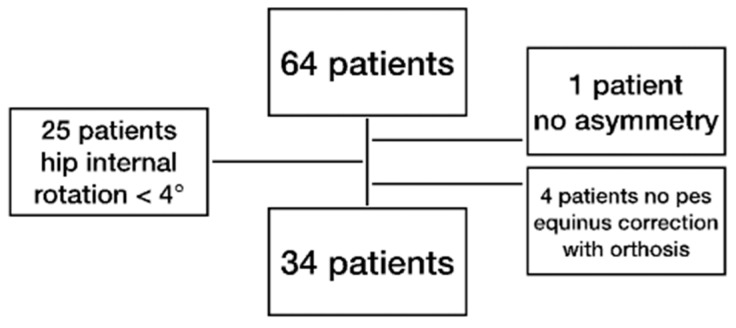
Schematic illustration of the inclusion of the patients. Middle row: number of patients; right and left row: number of and reason for excluded patients.

Gait analysis data from children with unilateral spastic cerebral palsy and spastic hemiplegia of other causes and equinus foot deformity were investigated in this study. Furthermore, these patients needed to have orthotics for the management of equinus deformity. The exclusion criteria were the absence of dynamic pelvic or hip asymmetry, botulinum toxin therapy of the leg conducted within three months prior to gait analysis, or a history of selective dorsal rhizotomy (See Figure 1). None of the children had a previous derotating femoral osteotomy. The inclusion criteria were the neurological disorder due to cerebral palsy, post-stroke, post-trauma, neoplasia, syndrome or post-infection, between 4 and 18 years old, and Gross Motor Function Classification System (GMFCS) I or II. Further inclusion criteria based on the gait analysis parameters were pes equinus of the affected side (ankle plantar flexion at IC and/or < 5° ankle dorsiflexion in the single support phase); ≥4° transversal hip internal rotation (>1 standard deviation (SD) of typically developing reference group); and ≥5° asymmetry of the hip or pelvis (>1 SD of typically developing reference group). Only one gait analysis per patient was included. The examination was carried out barefoot and with orthotic support of the equinus foot. The type of orthosis was based on the individual need of the patient and varied from heel wedges, insoles, and foot drop bandages to ankle–foot orthosis and supramalleolar orthosis, according to Nancy Hilton. All orthotics shared the goal of correcting the equinus position during walking to reduce the effects of increased plantar flexion on proximal segments and planes.

### 2.2. Measurements

All 3D gait analyses were carried out by the same experienced physiotherapists and pursuant to standardized procedures [25]. The participants were asked to walk along a 10-m walkway at a self-selected speed. Two gait analyses were performed in succession: the first barefoot and the second wearing their individual orthosis. Reflective markers were transferred from the foot to the orthosis according to a standardized protocol. At least three valid strides per patient and per condition were analyzed. A clinical examination was carried out as a standard part of each gait analysis. As a part of the clinical examination, femoral anteversion was measured using the TPAT (trochanteric prominence angle test).

### 2.3. Data Analysis

Several parameters for pelvic, hip, and ankle motion were derived from the gait analysis data. The pelvic motion in the transverse plane was calculated over the entire gait cycle (GC) due to the interdependent motion of the left and right pelvic hemispheres. Hip rotation was calculated as a mean during the stance phase (ST) and specifically at initial contact (IC). Furthermore, the internal hip rotational moments were calculated and used for further analysis. The ankle joint position was calculated at IC and as a mean during the stance phase. Furthermore, manually measured femoral torsion was used to determine a possible influence on pelvic and hip asymmetry. To determine any possible influences of the pes equinus and the femoral torsion on the pelvic and hip movements, differences in the joint angles between barefoot and orthotic management were calculated (See Table 1). They represent the change in joint angles while wearing the orthoses.

**Figure 2:**
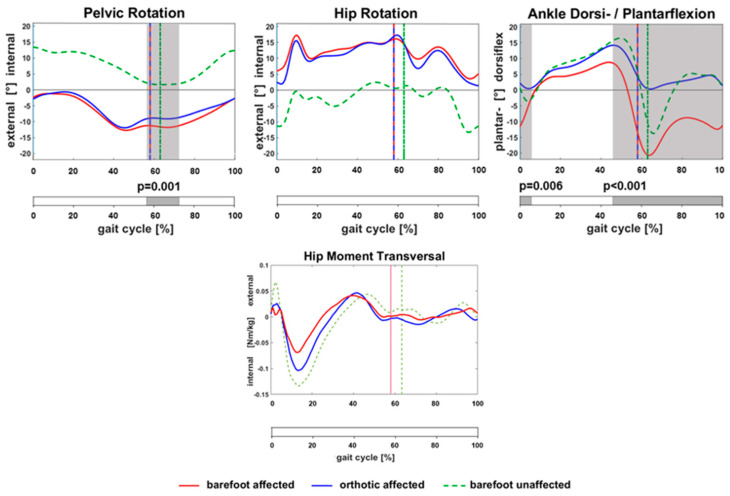
Statistic parametric mapping of kinematics of pelvic rotation, hip rotation, ankle flexion, and hip movement during gait. Grey area: significant changes on affected side between barefoot (red curve) and walking wearing orthoses (blue curve). The kinematics of the unaffected side (green curve) are shown as a reference.

**Figure 3:**
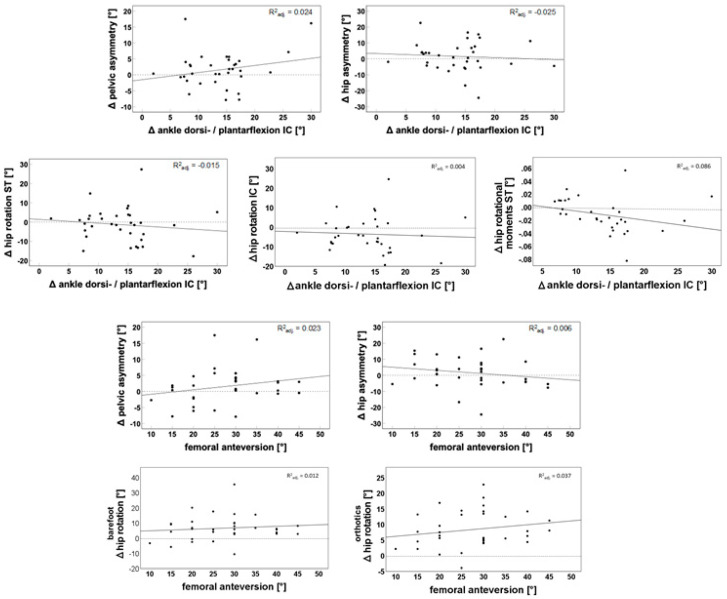
Linear regression of femoral anteversion or changes in pes equinus on changes in pelvic and hip asymmetry, hip rotation, and hip rotational moments. Δ pelvic asymmetry = barefoot affected side/orthotic affected side; Δ hip asymmetry = barefoot affected side/orthotic affected side; Δ hip rotation ST = orthotic affected side/barefoot affected side; Δ hip rotation IC = orthotic affected side/barefoot affected side; Δ hip rotational moments ST = orthotic affected side/barefoot affected side; Δ ankle dorsi/plantar flexion IC = orthotic affected side/barefoot affected side; barefoot Δ hip rotation = hip rotation 30%/hip rotation IC; orthotics Δ hip rotation = hip rotation 30%/hip rotation IC; R^2^_adj_ = R^2^ adjusted. Dotted line: zero line; continuous line: regression line.

To study the dynamic effect of equinus position over the first half of the stance phase (30%) on the hip rotation angle (See Figure 2), the Δ hip rotation was calculated with and without orthotics (See Figure 3). This difference was then correlated with the femoral anteversion angle to evaluate whether patients with a high anteversion angle showed significantly less Δ hip rotation during this phase.

### 2.4. Statistical Analysis

The data were tested for normal distribution using the Shapiro–Wilk test. Based on normally distributed data, for each parameter, descriptive statistics were calculated with means and SD. Paired sample *t*-tests and statistical parametric mapping (SPM), including the Bonferroni method, were used to investigate differences between barefoot and orthotic management. The influence of Δ ankle dorsi/plantar flexion IC and femoral anteversion on Δ pelvic and hip asymmetries and Δ hip rotation ST were analyzed by linear regression analyses. The significance level was set at α ≤ 0.05. 

### 2.5. Sample Size

Due to a retrospective study design, out of 64 patients with unilateral spastic cerebral palsy and spastic hemiplegia of other causes who underwent a gait analysis, 34 were included in this study, according to the inclusion and exclusion criteria (See Figure 1).

## 3. Results

Sixty-four children with unilateral spastic cerebral palsy and spastic hemiplegia of other causes were found in our database. A total of thirty patients were excluded due to the inclusion and exclusion criteria from the gait analysis data.

The selection process resulted in 34 children (17 females, 17 males) with unilateral spastic (*n* = 24) or spastic-dystonic (*n* = 10) hemiplegia of different causes. Reasons for the movement disorder were cerebral palsy (*n* = 24), post-stroke (*n* = 6), post-traumatic (*n* = 1), neoplastic (*n* = 1), syndromal (*n* = 1), and post-infectious (*n* = 1) disease. Twenty-seven patients had GMFCS-level I and 7 GMFCS-level II. The patients’ mean age at examination was 10 ± 4 years (4 to 17 years old). Individual orthotic management was used for pes equinus correction and compensation for leg length discrepancy. In the clinical examination, the participants showed a femoral anteversion from 10° to 45° (mean = 27.65° ± 9.07°).

Pelvic and hip asymmetry did not significantly change with orthoses (each by approx. 2°) (See Table 2). There was a significant difference in mean pelvic rotation during GC. With orthotic management, the pelvis rotated approx. 1° more inwards. When walking barefoot, the group showed an ankle plantar flexion at IC of around 12°, which was significantly reduced with orthoses (by approx. 14°) and resulted in a mean dorsiflexion at IC around 2°.

With orthotic equinus management, there was a significant pelvic internal rotation at the end of ST and the beginning of the swing phase compared to barefoot (See Figure 2). There was a visible but not significant deviation of hip rotation with orthoses compared to barefoot at IC but no longer in midstance (MST). Hip moments during ST showed no significant changes. Pes equinus was corrected at IC, the end of ST, and during the swing phase using orthoses. 

The changes in the ankle position at IC did not relate to the changes in pelvic asymmetry (β = 0.222, *p* = 0.189), hip asymmetry (β = −0.128, *p* = 0.662), Δ hip rotation at ST (β = −0.195, *p* = 0.486) and at IC (β = −0.096, *p* = 0.731), or Δ hip rotational moments at ST (β = −0.001, *p* = 0.109) (See Figure 3). Femoral anteversion did not show a relationship with rotational asymmetries (pelvis: β = 0.139, *p* = 0.190) (hip: β = −0.196, *p* = 0.280). The changes in the hip rotation at IC to MST did not relate to the femoral anteversion. This could be shown with orthoses (β = 0.124, *p* = 0.278) and barefoot (β = 0.98, *p* = 0.542).

**Figure 4:**
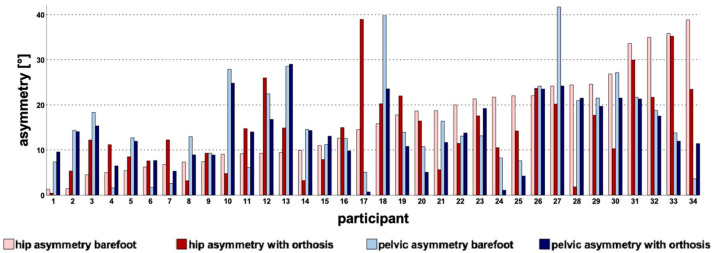
Illustration of hip (red) and pelvic (blue) asymmetry barefoot (light colored) and with orthosis (dark colored). X-axis: 34 patients (4 bars per participant), in order of ascending hip asymmetry barefoot. Y-axis: absolute value of difference in affected and unaffected sides.

The hip asymmetry increased in 15 participants and decreased in 19 participants while wearing their orthosis (See Figure 4). Twenty-three children showed a reduced pelvic asymmetry when wearing the orthosis, while 11 children had an increased pelvic asymmetry with the orthosis.

## 4. Discussion

The current study investigated the influence of orthotic equinus correction in children with unilateral spastic cerebral palsy and spastic hemiplegia of other causes on hip and pelvic rotation and associated asymmetries. All the participants showed a significant reduction in plantar flexion when wearing orthotics, underlining that there was a functional correction of pes equinus during gait and corroborating the findings of previous investigations [14,16,17,26].

However, this correction of pes equinus showed variable effects on hip and pelvic rotation, even though pes equinus has been associated with pelvic retraction and internal hip rotation in the previous literature [10,27]. From this global evaluation, the clinical relevance of these effects seems only to be relevant for some children, or other factors mask this association.

On average, only a significant difference in mean pelvic rotation at the end of the stance phase and the beginning of the swing phase of 1° toward the internal rotation could be found, which we consider not to be clinically relevant. This reduction could be explained by the reduction in leg extension due to the orthosis and, therefore, the reduction in pelvic retraction movement and the lifting of the foot is no longer delayed. According to Aminian et al., the retraction on the affected side was also seen as compensation for the stride length of the opposite side, which is impaired by the weakness of the hip extensors on the affected side [28].

There was a trend in the linear regression analysis that when the pes equinus was corrected while wearing orthoses, there was a marginal decrease in the hip asymmetry, and the asymmetry of the pelvis slightly increased. The hip rotation at initial contact and hip rotational moments during the stance phase showed no significant relationship with the ankle position. Furthermore, the amount of femoral anteversion did not significantly affect rotational asymmetries when wearing an orthosis. However, there was also a slight opposite trend as the femoral anteversion increased, the pelvic asymmetry also increased, and the hip asymmetry decreased (See Figure 3).

In line with the results of Brunner et al. [5], Figure 2 shows a visible but not significant deviation of hip rotation with orthoses compared to barefoot at initial contact, but no longer during midstance. This may explain why the dynamic effect is most relevant during the early stance phase or why static torsional or lever arm preconditions are the more relevant factors for torsional positioning after the loading response.

For a better understanding, we additionally correlated the dynamic change in hip rotation during the first 30% of the gait cycle with the clinically measured femoral anteversion angle (See Figure 3). There, we could not find a significant relationship either with orthoses or barefoot. This may have several explanations. First, if there is remaining passive internal rotation in the hip despite an increased femoral anteversion, the dynamic effects of equinus foot position on hip rotation may still work. Another explanation may be that other dynamic effects could be relevant and mask the dependence between this suggested effect. Furthermore, clinical evaluation of the femoral anteversion angle shows only a weak correlation with the anatomical anteversion, which may also mask this effect. This will be further addressed in the limitations section.

Figure 4 further illustrates that there was a very variable effect of the orthoses on the rotational asymmetries. About half of the patients saw improved hip asymmetry, and about two-thirds saw improved pelvic asymmetry (See Figure 4). However, the effects on asymmetries were highly inconsistent, and possible reasons need to be further discussed.

Weak hip abductors [29] or increased hip flexion, which contribute to an increase in the internal rotating movement of the hips, may also play a role [9]. There is some limited evidence that soft tissue surgery of the hip flexors and adductors, in addition to femoral derotational osteotomy, improves pelvic retraction, as well as internal hip rotation [30].

A potential leg length difference was compensated with orthotic management. The influence of leg length differences on rotational asymmetries is possible; however, to conclude this, the exact calculation would be beneficial. Furthermore, malalignment, altered tibial torsion, and a structural or functional pointed foot may influence a possible correction of the asymmetries.

In summary, we recommend a correction of the pes equinus through orthoptic management for a proven significant reduction in ankle plantar flexion and, thus, functional correction. Multiple factors seem to influence rotational asymmetry. Our results underline that other factors need to be considered to understand internal rotation hip and pelvic retraction patterns in children with unilateral spastic cerebral palsy and spastic hemiplegia of other causes. We suggest that a more comprehensive dynamic and static investigation is needed, taking into account various additional confounding factors, such as tibia torsion, muscular weaknesses, and joint contractures, in order to improve our understanding of the mechanisms underlying rotational asymmetries. Since internal rotation is one of the most disturbing aspects of gait impairment in these children, it is crucial to inform the children and their parents that the internal rotation of the hip and pelvic asymmetry may persist despite orthotic management of the equinus foot position and potentially needs to be addressed by other means, such as rotational orthotics or surgical correction through rotational osteotomy.

There are several limitations to mention. One reason for a pronounced internal rotation gait is increased femoral anteversion. This was manually measured in the current study with the commonly used Craig’s test. Measured in the prone position, the hip is rotated until the greater trochanter can be palpated most prominently. The amount of torsion corresponds to the angle of the flexed lower leg to the vertical. Several studies have compared the widely used Craig’s test and computed tomography to measure femoral anteversion without significant correlation [31,32]. Therefore, the analysis of whether a higher femoral anteversion produces a smaller effect on correcting asymmetry in walking is limited. However, in our study, the differences in gait parameters between barefoot and wearing orthoses were the main parameters not affected by femoral anteversion measurement.

The type of orthosis was based on the individual need of the patient. Due to the individual orthotic management, it was not possible to create subgroups of orthotic type. However, to answer the main question, it was only relevant that the orthosis corrected the equinus foot functionally, which was the case in all participants.

Our retrospective study population included children with spastic and spastic–dystonic hemiplegia with variable causes, which resulted in a rather inhomogeneous population. For a more detailed analysis investigating further possible influencing factors, such as further stratification, e.g., on the influence of spastic or dyskinesia (dystonia) or on the influence of structural or functional pes equinus—a larger number of patients should be included.

All participants walked first barefoot and then with orthoses. The changes in gait pattern between the two examinations could have occurred due to muscular and/or mental fatigue. No studies have investigated the effect of fatigue on physical function. A prospective study randomizing the order of walking with a larger sample size could outweigh some of the limitations of the current study.

The variability of dynamic effects of orthotic management on the hip and pelvic rotation, which was found in our study, clearly shows that there is a need to further investigate these mechanisms. A major limitation of this retrospective approach is the limited number of patients, which did not allow for further subgroup analysis.

## 5. Conclusions

Orthotic management of pes equinus significantly reduced ankle plantar in children with unilateral spastic cerebral palsy or spastic hemiplegia of other causes and is, therefore, recommended for functional correction. However, there was a variable effect on the hip and pelvic asymmetry. The increased hip internal rotation problem appears to be multifactorial. Children and their parents need to be informed that rotational asymmetries may persist when using orthoses.

## Figures and Tables

**Table 1:** Description of the calculation of the used variables.

Variable	Calculation
Pelvic asymmetry barefoot [°]	|mean pelvic rotation GC affected/unaffected side|
Pelvic asymmetry with orthosis [°]	|mean pelvic rotation GC affected/unaffected side|
Δ pelvic asymmetry [°]	barefoot affected side/orthotic affected side
Hip asymmetry barefoot [°]	|mean hip rotation ST affected/unaffected side|
Hip asymmetry with orthosis [°]	|mean hip rotation ST affected/unaffected side|
Δ hip asymmetry [°]	barefoot affected side/orthotic affected side
Δ mean hip rotation ST [°]	orthotic affected side/barefoot affected side
Δ hip rotation IC [°]	orthotic affected side/barefoot affected side
Δ hip rotational moments ST [°]	orthotic affected side/barefoot affected side
Barefoot Δ hip rotation [°]	hip rotation midstance/hip rotation initial contact
Orthotic Δ hip rotation [°]	hip rotation midstance/hip rotation initial contact
Δ ankle dorsi/plantar flexion IC [°]	orthotic affected side/barefoot affected side

Δ = delta; | | = absolute value; GC = gait cycle; ST = stance phase; IC = initial contact.

**Table 2:** Comparison of kinematic gait data barefoot and with orthoses.

Parameter	Barefoot (Mean ± SD) [°]	Orthoses (Mean ± SD) [°]	*p*-Value
Pelvic rotation GC (mean)	−7.4 ± 5.5	−6.1 ± 4.7	0.018
Pelvic rotation GC (ROM)	16.6 ± 4.3	16.3 ± 4.9	0.657
Pelvic asymmetry	15.5 ± 9.8	13.9 ± 7.2	0.106
Hip rotation ST (mean)	12.9 ± 6.3	11.6 ± 8.4	0.398
Hip rotation ST (ROM)	21.1 ± 8.2	25.1 ± 9.4	<0.001
Hip asymmetry	16.2 ± 10.2	14.6 ± 9.3	0.326
Ankle dorsi/plantar flexion IC	−11.5 ± 6.0	2.2 ± 4.1	<0.001
Ankle dorsi/plantar flexion ST (mean)	3.2 ± 8.8	8.0 ± 4.6	<0.001

GC: gait cycle, ROM: range of motion, ST: stance phase, IC: initial contact. Pelvic rotation: protraction = +/retraction = −; hip rotation: internal = +/external = −; pelvic asymmetry = |affected/unaffected pelvic rotation GC (mean)|; hip asymmetry = |affected/unaffected hip rotation ST (mean)|; ankle: dorsiflexion = +/plantar flexion = −.

## Data Availability

All data are stored at the University Children’s Hospital Zurich.
